# Incidence and development of validated mortality prediction model among asphyxiated neonates admitted to neonatal intensive care unit at Felege Hiwot Comprehensive Specialized Hospital, Bahir Dar, Northwest Ethiopia, 2021: retrospective follow-up study

**DOI:** 10.1186/s12887-024-04696-0

**Published:** 2024-03-28

**Authors:** Yibeltal Shitu Tegegne, Tilahun Yemanu Birhan, Habtamu Takele, Fantahun Ayenew Mekonnen

**Affiliations:** 1 Department of Epidemiology, Curative and Preventive Health Service, Amhara Regional Health Bureau, Bahir Dar, Ethiopia; 2 Department of Epidemiology and Biostatistics, Institute of Public Health, College of Medicine and Health Sciences, University of Gondar, Gondar, Ethiopia; 3 Department of Public Health, College of Health Sciences, Debre Markos University, Debre Markos, Ethiopia

**Keywords:** Asphyxia, Neonate, Mortality, Risk score, Ethiopia

## Abstract

**Introduction:**

Perinatal asphyxia is failure to maintain normal breathing at birth. World Health Organization indicates that perinatal asphyxia is the third major cause of neonatal mortality in developing countries accounting for 23% of neonatal deaths every year. At global and national level efforts have done to reduce neonatal mortality, however fatalities from asphyxia remains high in Ethiopia (24%). And there are no sufficient studies to show incidence and prediction of mortality among asphyxiated neonates. Developing validated risk prediction model is one of the crucial strategies to improve neonatal outcomes with asphyxia. Therefore, this study will help to screen asphyxiated neonate at high-risk for mortality during admission by easily accessible predictors. This study aimed to determine the incidence and develop validated Mortality Prediction model among asphyxiated neonates admitted to the Neonatal Intensive Care Unit at Felege-Hiwot Comprehensive Specialized Hospital, Bahir Dar, Ethiopia.

**Method:**

Retrospective follow-up study was conducted at Felege-Hiwot Comprehensive Specialized Hospital from September 1, 2017, to March 31, 2021. Simple random sampling was used to select 774 neonates, and 738 were reviewed. Since was data Secondary, it was collected by checklist. After the description of the data by table and graph, Univariable with *p*-value < 0.25, and stepwise multivariable analysis with *p*-value < 0.05 were done to develop final reduced prediction model by likelihood ratio test. To improve clinical utility, we developed a simplified risk score to classify asphyxiated neonates at high or low-risk of mortality. The accuracy of the model was evaluated using area under curve, and calibration plot. To measures all accuracy internal validation using bootstrapping technique were assessed. We evaluated the clinical impact of the model using a decision curve analysis across various threshold probabilities.

**Result:**

Incidence of neonatal mortality with asphyxia was 27.2% (95% CI: 24.1, 30.6). Rural residence, bad obstetric history, amniotic fluid status, multiple pregnancy, birth weight (< 2500 g), hypoxic-ischemic encephalopathy (stage II and III), and failure to suck were identified in the final risk prediction score. The area under the curve for mortality using 7 predictors was 0.78 (95% CI 0.74 to 0.82). With ≥ 7 cutoffs the sensitivity and specificity of risk prediction score were 0.64 and 0.82 respectively.

**Conclusion and recommendation:**

Incidence of neonatal mortality with asphyxia was high. The risk prediction score had good discrimination power built by rural residence, bad obstetric history, stained amniotic fluid, multiple pregnancy, birth weight (< 2500 g), hypoxic-ischemic encephalopathy (stage II and III), and failure to suck. Thus, using this score chart and improve neonatal and maternal service reduce mortality among asphyxiated neonates.

**Supplementary Information:**

The online version contains supplementary material available at 10.1186/s12887-024-04696-0.

## Introduction

Perinatal asphyxia (PNA) is an insufficiency of blood flow and oxygen to the fetus from the placental side or the fetus’s pulmonary Origen. It is also defined as failure to maintain normal breathing at birth [1–3]. Perinatal asphyxia(PNA) is a common and serious global life-threatening neonatal problem [4]. It is a significant cause of acquired brain injury occurring in the neonatal period [5].

According to the research done in 2016 on six Low and Middle Income Countries (LMICs), fetal asphyxia was 46.6% [6]. In 2017, the incidence of PNA in developed countries is 2 per 1000 live births, but the rate is 10 times greater in developing countries where the setting with low quality and limited access to maternal and neonatal service delivery systems [7].

WHO indicates that PNA is the third major cause of neonatal mortality next to sepsis and preterm births accounting for an estimated 23% (4 million) of neonatal deaths every year in developing countries [8]. It is also the 5th most frequent cause of death among under-five children next to prematurity, infection, birth injury, and tetanus [9]. PNA is responsible for 42 million disability-adjusted life years [10]. In the neonatal period, 15–20% of asphyxiated neonates will die and about 25% of survivors will have serious neurological sequels, such as cerebral palsy, mental retardation, and epilepsy, leading to detrimental long-term consequences for both child and family [7].

Another multi-country prospective cohort study conducted on 11 communities in South Asia and sub-Saharan Africa(SSA) (2012–2016) reported that the most common causes of neonatal mortalities were perinatal asphyxia 40% and 34% in South Asia and SSA respectively [11].

According to the 2014 WHO report for Ethiopia, among the direct causes of under-five mortality, 14% of the death was accounted by asphyxia [12]. Early neonatal death was caused by many causes, including perinatal asphyxia (34%), prematurity (25%), sepsis, and other infectious diseases (18%) [13, 14]. WHO report (2015) also indicates that the main causes of neonatal deaths in Ethiopia were birth asphyxia (31.6%), prematurity (21.8%), and sepsis (18.5%) (18). Approximately 70–80% of these neonatal deaths are triggered by preventable and treatable conditions with easily accessible and inexpensive treatments [15]. Intrapartum asphyxia accounts for 814,000 deaths worldwide [15]. Perinatal asphyxia, due to lack of a standard feto-neonatal fluid and oxygenation resuscitation in peripartum, at birth, and in the first minutes of life is a sensitive measure of the quality of care given in the perinatal period, both to the pregnant woman and the newborn, with high potential for prevention of death through early diagnosis and treatment [16]. Preconception, antepartum, intrapartum, and postpartum risk factors have been associated with the case fatality of asphyxia. Just different review showed that asphyxia is primarily antepartum in origin in 50% of cases, intrapartum in 40%, and postpartum in the remaining 10% of cases [17]. Guidelines and management protocols show that predictors related to neonatal mortality with asphyxia are summarized into five thematic areas: Socio-demographic, antepartum, intrapartum, neonatal-related, and clinical factors [1, 12, 18].

Predictive modeling is aimed at developing tools that can be used by health professionals to predict the probability of the occurrence of an event. There has been a huge increase in the popularity of developing tools for the prediction of outcomes at the level of the individual patient. The advantage of prediction models is, that they formally combine risk factors, allowing for more accurate risk estimation [19, 20]. Predicting high-risk neonates is vital for determining for public health policy decisions and management of pregnancy, childbirth, and neonatal periods, including the appropriate selection of prognostic risk factors and readmes of selective care pathways for high-risk asphyxiated neonates and pregnancies [21]. Furthermore, early identification of neonates at risk of mortality can enable health care providers for early treatment, which has a direct impact on their survival and morbidity [22].

Our objective is to develop a risk assessment tool for neonatal mortality with asphyxia that would include Socio-demographic, antepartum, intrapartum, neonatal-related, and clinical factors from a retrospective neonatal record data.

Globally efforts have done to reduce neonatal mortality, including mortality among perinatal asphyxia, from 5.1 million in 1990 to 2.6 million in 2016. The decrement of 49% is, however, slower than the rate of decline in children aged 1 to 59 months (62%). SDG develop the strategy, by 2030, to end preventable deaths of newborns and aimed at reducing neonatal mortality at least as low as 12 per 1,000 live births in all countries [23].

Ethiopia's Ministry of Health also have been working for years to improve newborn and child survival rates by making health services available and establishing Guidelines for women and children to treat asphyxiated neonates at birth [24]. Even though Ethiopia met its goal of reducing child mortality two years earlier than the set target of millennium development goal 4 (MDG4), Fatalities from perinatal asphyxia remain high [12, 25].

At global and national level efforts have done to reduce neonatal mortality, however fatalities from asphyxia remains high in Ethiopia (24%). And there are no sufficient studies to show incidence and prediction of mortality among asphyxiated neonates. Developing validated risk prediction model is one of the crucial strategies to improve neonatal outcomes with asphyxia. Therefore, this study will help to screen asphyxiated neonate at high-risk for mortality during admission by easily accessible predictors. This study aimed to determine the incidence and develop validated Mortality Prediction model among asphyxiated neonates admitted to the Neonatal Intensive Care Unit at Felege-Hiwot Comprehensive Specialized Hospital, Bahir Dar, Ethiopia.

## Methods and materials

### Study setting

The study was conducted at Felege Hiwot Comprehensive Specialized Hospital (FHCSH). FHCSH is one of the largest hospitals in Ethiopia which is found in Bahir Dar City, the capital of Amhara National Regional State, North West Ethiopia. It is 575KM far from Addis Ababa. FHCSH was purposely selected with fact that it is the center and biggest high-level referral hospital in the region visited by around seven million people per year from the surrounding zones and nearby regions.

Currently, the hospital has been established from clinical and non-clinical departments /service units / to provide diagnostic, curative & rehabilitation services at outpatient &inpatient units. It also provides disease prevention & health promotion services. NICU is a unit under the pediatrics and child health department that has 38 nurses, 6 general practitioners, 2 Senior Physicians staff, and 83 beds to provide an inpatient medical service for neonates within 28 days of age.

### Study design and period

The Institutional based retrospective follow-up study was conducted to determine the incidence and develop a validated risk prediction model for mortality among asphyxiated neonates admitted to NICU from September 1, 2017, to March 31, 2021, at FHCSH Bahir Dar, Ethiopia.

#### Population

All under 28 days of age neonates with perinatal asphyxia admitted to NICU at FHCSH were the source population. Source population were not to our study periods (September 1, 2017, to March 31, 2021). Whereas Study population: All neonates with perinatal asphyxia admitted to NICU at FHCSH from September 1, 2017, to March 31, 2021, during the study period were the study population.

### Inclusion and exclusion criteria

All neonates with perinatal asphyxia who were admitted and registered in NICU at FHCSH from September 1, 2017, to March 31, 2021, during the study period were included.

Thirty-six asphyxiated neonates with incomplete information on outcome status, date of admission, and date of discharge in the medical records were excluded.

#### Pilot study

A pilot study is important to obtain preliminary data for the outcome Variable and assess and select easily accessible predictors [26, 27]. As far as our knowledge, there is scarce of information in Ethiopia about the incidence and prognostic factor of mortality among asphyxiated neonates in the prediction model. Therefore to overcome this we performed a pilot study on 74 [27] Samples at FHCSH before the actual study was conducted. The incidence of Mortality among asphyxiated neonates was 22(29.7%). Then, we used this result to calculate sample size, screen easily accessible predictors, and check the applicability checklist by data collectors.

### Sampling technique and sample size determination

In this study, secondary data from FHCSH NICU register log-book and patient folder were collected from September 1, 2017, to March 31, 2021. Patients’ medical registration number was used as a sampling frame. From the total of 1172 registered asphyxiated neonates in the four-year time interval 774 neonates were selected by a simple random sampling technique and 738 were reviewed.

### The sample size for the prognostic prediction model

The sample size for the prediction model was calculated Considering 10 events per predictor parameter [19]. $n=\frac{{\text{P}}*10}{{\text{P}}(\mathrm{\%}) }=\frac{23*10}{29.7\mathrm{\% }}=774$, n is sample size = 774, P is parameter = 23, P (%) is the probability of outcome = 29.7% taken from the pilot study and 23 predictors were selected after the pilot study by assessing the accessibility and easy applicability of each predictor.

### Study variables

The dependent variable was Mortality among Asphyxiated Neonates. Prognostic factors are Socio-demographic, antepartum, intrapartum, clinical, and neonatal-related factors. Residence, bad obstetric history, amniotic fluid status, multiple pregnancy, birth weight (< 2500), hypoxic-ischemic encephalopathy (stage II and III) and failure to suck have remained as powerful predictors of neonatal mortality with PNA in the final reduced model.

### Data collection technique and quality assurance

**Fig. 1:**
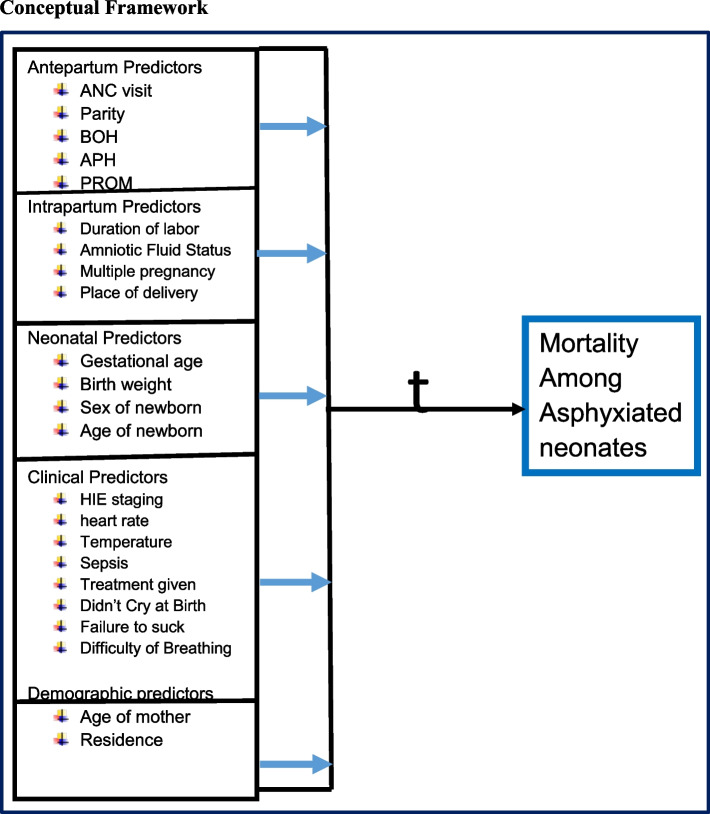
Conceptual framework showing the relation between mortality among asphyxia and its predictors (Source: “A conceptual framework for prognostic research” [28] After revising related literature and chats [18, 29–32]) ANC = antenatal care, BOH = bad obstetric history, APH = antepartum hemorrhage, PROM = premature rupture of membrane, HIE = hypoxic-ischemic encephalopathy, t = follow-up time until outcome happens in prognostic research

Data was collected by a data extraction checklist/tool which is developed by the principal investigator after reviewing the NICU log book register, patient folder, and different kinds of related literature (Fig. 1). Before data collection, the records were reviewed and cards of asphyxiated neonates were identified by their medical registration/card number from the NICU log book register.

Data were collected by 2 BSC nurses and supervised by MPH students after training and orientation were given. Collected data were checked for completeness and consistency on each day of data collection by the principal investigator and supervisor.

### Data management

The data were checked for completeness and entered into Epi-Info v 7.2.4. Then we exported to the R statistical software version 4.1.0 for further analysis and modeling.

#### Missing data

Missing data were imputed using the ‘multiple imputations. We assessed the pattern of missing data graphically by md. Pattern and xplot function in R Software version 4.1.0 to show the distribution of observed and missed which were missing at a random pattern, and we, therefore, performed a multivariate imputation by chained equations using the “mice” package in R. Missing results were imputed for all variables evaluated in the prediction model, but not for “Mortality of Perinatal Asphyxia” because we analyzed only neonates who have discharge summary for outcome status. Linear regression was used to predict a scale variable and logistic regression to predict categorical variables. We imputed missing values with the mode for categorical data or the median for continuous data. Among 23 predictors, Duration of labor 78 (10.6%), PROM 38(5.1%), APH 37(5.0%), and gestational age 35(4.7%) were the most common predictors showing missing values (ANNEX VI).

### Method of statistical analysis

#### Model development, performance, calibration, validation, risk score development and decision curve analysis

##### Model development

To investigate the association between each predictor and mortality among asphyxiated neonates’ univariable logistic regression was performed. To be more liberal, we fitted all the predictors at a cut-off point of *p*-value < 0.25 in the univariable analysis to select prognostic factors for multivariable analysis. Then, we used a stepwise backward elimination strategy with a *p*-value < 0.15 [33, 34] for the likelihood ratio test to fit the final reduced model. Results from the univariable and multivariable logistic regression models were expressed in terms of the beta coefficients with 95% confidence intervals.

##### Model performance

Model Discrimination power was assessed by Receiver Operating Characteristic (ROC) curve, Area under the Curve (AUC), sensitivity, and specificity. AUC of 0·5 indicates the chance or no predictive ability, 0.7 is acceptable predictive ability and 1 is perfect predictive ability.

##### Model calibration

The goodness of Model-fitness was checked by Hosmer–Lemeshow and a calibration plot both graphically and statistically to compare observed and predicted risk probability.

##### Model validation

Original Beta coefficients of regression with 95% confidence intervals and AUC of the model were internally validated using the bootstrapping technique. To measure the reproducibility of predicted outcome, many scholars have been using 10,000 randomly resampled study participants [35]. To do these, 10,000 random bootstrap samples with replacement were drawn from the data set with complete data on all predictors [36]. Bias-corrected AUC, Risk score, Sensitivity, and Specificity were calculated. The model’s predictive performance after internal validation is expected to be the performance that can be applied to similar settings and populations in the future.

##### Risk score development

To create an easily applicable and clinically useful Mortality prediction Score among asphyxiated neonates, we changed significant coefficients from the multivariable model to a rounded integer by dividing each significant coefficient by the lowest significant coefficient. We determined the total score for each individual by adding up the assigned points in each significant predictor. The probability of predicted risk of mortality was presented by three (low, medium, and high) categories of the risk score just for practical applicability.

The score was changed to a dichotomous “prediction test,” allowing each asphyxiated neonate to be classified as having a high or low risk of mortality. We calculated sensitivity specificity positive and negative predictive values, and the likelihood ratios of categorized values around different cutoff points of 5, 6, 7, 8, and 9.

##### Decision curve analysis

To assess the clinical and public health impact of the model, decision curve analysis (DCA) was carried out, by threshold probabilities (0 to 1) versus standardized net benefit. In the DCA, the model was compared against three scenarios; intervention based on score chart, “intervention for all” and “none of intervention”. In this study, the intervention was considered to improve critical care services including therapeutic hypothermia for high-risk asphyxiated neonates.

##### Ethical consideration

The study was conducted among asphyxiated neonates admitted to Neonatal Intensive Care Unit from September 1, 2017, to March 31, 2021, at FHCSH Bahir Dar, Ethiopia. Ethical clearance and a Letter of cooperation were obtained from the Institutional Review Committee from the University of Gondar, College of Medicine and health sciences with (Ref No./IPH/1475/2013). Felege-Hiwot Compressive Specialized Hospital was informed about the study objective through a written letter. Permission was obtained from the medical director of the hospitals. Head of Neonatal Intensive Care Unit had been waived to have data from the medical records of Neonate used in research. All methods were carried out in accordance with relevant guidelines and regulations. Confidentiality had been maintained at all levels of the study.

## Result

### Distribution of maternal demographic, antepartum and intrapartum predictors of mortality among asphyxiated neonates

A total of 738 charts of neonates admitted with perinatal asphyxia (PNA) in the NICU were reviewed. Out of 738 mothers, 595(80.6%) having neonates admitted to NICU with PNA was found between the age groups of 20–35 years. Of the total of 201 death among asphyxiated neonates, more than three quarters (77.6%) were found in the 20–35 years of age group. Concerning the place of residence 112(55.7%) mortality was assessed in rural.

The majority of mothers 707 (95.8%) had a history of antennal care follow-up. Of the total of survived, and died neonates 54.2% and 50.8% belong to Primiparous mothers respectively. Of 738 mothers with Neonatal admission due to PNA 138(18.7%) and 127(17.2%) had bad obstetric history and PROM orderly.

Out of the study groups, mothers who had a duration of labor > 18 h belong to 110 (20.5%) in Survived and 43(21.4%) in died neonates respectively. 234 (43.6%) out of the survived and 117 (58.2%) out of died neonates, mothers had a history of stained amniotic fluid at delivery. Concerning the type of pregnancy, 13(2.4%) mothers from out of survived, and 14 (7.0%) from died neonates had multiple pregnancy. While almost half of survived (53.4%) and (54.2%) died neonates were referred to this hospital (Table 1).

**Table 1:** Distribution of maternal demographic, antepartum, and intrapartum predictors of mortality with PNA admitted to NICU at FHCSH, Bahir Dar, Ethiopia, 2021

Demographic Predictors	Category	Outcome status of PNA		Total n (%)
		Survived n (%)	Died n (%)	
**Maternal age in Years**	20–35	439 (81.8)	156 (77.6)	595 (80.6)
	< 20	78 (14.5)	30 (14.9)	108 (14.6)
	> 35	20 (3.7)	15 (7.5)	35 (4.8)
**Place of Residence**	Urban	326 (60.7)	89 (44.3)	415 (56.2)
	Rural	211 (39.3)	112 (55.7)	323 (43.8)
**Antepartum Predictors**				
**Antenatal Care visit**	Yes	515 (95.9)	192 (95.5)	707 (95.8)
	No	22 (4.1)	9 (4.5)	31 (4.2)
**Parity**	Multipara	202 (37.6)	68 (33.8)	270 (36.6)
	Primipara	291 (54.2)	102 (50.8)	393 (53.2)
	Grand Multipara	44 (8.2)	31 (15.4)	75 (10.2)
**Bad Obstetric History**	No	449 (83.6)	151 (75.1))	600 (81.3)
	Yes	88 (16.4)	50 (24.9)	138 (18.7)
**Antepartum hemorrhage**	No	495 (92.2)	184 (91.5)	679 (92.0)
	Yes	42 (7.8)	17 (8.5)	59 (8.0)
**Premature ROM**	No	447 (83.2)	164 (81.6)	611 (82.8)
	Yes	90 (16.8)	37 (18.4)	127 (17.2)
**Intrapartum Predictors**				
**Duration of labor**	≤ 18 h	427 (79.5)	158 (78.6)	585 (79.3)
	> 18 h	110 (20.5)	43 (21.4)	153 (20.7)
**Status of Amniotic Fluid**	Clear	303 (56.4)	84 (41.8)	387 (52.4)
	Stained	234 (43.6)	117 (58.2)	351 (47.6)
**Type of Pregnancy**	Single	524 (97.6)	187 (93.0)	711 (96.3)
	Multiple	13 (2.4)	14 (7.0)	27 (3.7)
**Place of delivery**	Inborn	250 (46.6)	92 (45.8)	342 (46.3)
	Referred	287 (53.4)	109 (54.2)	396 (53.7)

### Distribution of neonatal and clinical predictors of mortality among asphyxiated neonates

58(10.8%) of 537 Survived and 37(18.4%) of 201 died neonates gestational age was less than 37 weeks (preterm) and out of 738 neonates with perinatal asphyxia 171(23.2%), 245(33.2%), and 481(65.2%) were birth weight < 2500(g), age of neonate > 6 h at admission and male sex respectively.

**Fig. 2:**
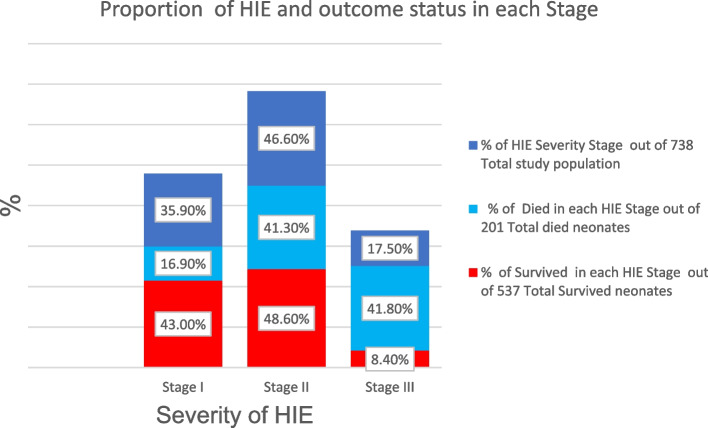
Proportion of hypoxic ischemic encephalopathy and outcome status in each stage among asphyxiated neonates admitted to NICU at FHCSH, Bahir Dar, Ethiopia, 2021

Approximately half of (46.6%) neonates with Hypoxic- Ischemic Encephalopathy (HIE) were found in stage II or moderate (Fig. 2). Of 738 neonates with PNA 593(80.4%) and 383(51.9%) had 100–160 heart rates, and 35.5–37.5^o^_c_ temperatures respectively.132 (65.7%) Out of died and 350(65.2%) from survived had sepsis. Almost all neonates admitted to NICU (98.5%) were given treatment during the admission period. Regarding the sign and symptoms of neonates at admission, from the total of 738 neonates with PNA 147(19.9%), 186(22.8%), and 183(24.4%) didn’t cry immediately at birth, failure to suck, and difficulty of breathing respectively (Table 2).

**Table 2:** Distribution of neonatal and clinical predictors of mortality with PNA admitted to NICU at FHCSH, Bahir Dar, Ethiopia, 2021

Neonatal Predictors	Category	Outcome status of PNA		Total n (%)
		Survived n (%)	Died n (%)	
Gestational Age	Term	453 (84.4)	151 (75.1)	604 (81.8)
	Preterm	58 (10.8)	37 (18.4)	95 (12.9)
	Posterm	26 (4.8)	13 (6.5)	39 (5.3)
Birth Weight(gram)	≥ 2500	431 (80.3)	136 (67.7)	567 (76.8)
	< 2500	106 (19.7)	65 (32.3)	171 (23.2)
Age of Neonate	≤ 6 h	362 (67.4)	131 (65.2)	493 (66.8)
	> 6 h	175 (32.6)	70 (34.8)	245 (33.2)
Sex of New born	Female	199 (37.1)	58 (28.9)	257 (34.8)
	Male	338 (62.9)	143 (71.1)	481 (65.2)
Clinical Predictors				
Heart Rate (bpm)	100–160	433 (80.6)	160 (79.6)	593 (80.4)
	< 100	15 (2.8)	11 (5.5)	26 (3.5)
	> 60	89 (16.6)	30 (14.9)	119 (16.1)
Temperature(^o^c)	35.5–37.5	298 (55.5)	85 (42.3)	383 (51.9)
	< 35.5	207 (38.5)	108 (53.7)	315 (42.7)
	> 37.5	32 (6.0)	8 (4.0)	40 (5.4)
Sepsis	No	187 (34.8)	69 (34.3)	256 (34.7)
	Yes	350 (65.2)	132 (65.7)	482 (65.3)
Treatment Given	Yes	527 (98.1)	200 (99.5)	727 (98.5)
	No	10 (1.9)	1 (0.5)	11 (1.5)
Didn’t cry immediately at birth	No	444 (82.7)	147 (73.1)	591 (80.1)
	Yes	93 (17.3)	54 (26.9)	147 (19.9)
Failure to suck at Admission	No	431 (80.3)	139 (69.2)	570 (77.2)
	Yes	106 (19.7)	62 (30.8)	168 (22.8)
Difficulty Breathing At presentation	No	415 (77.3)	140 (69.6)	555 (75.2)
	Yes	122 (22.7)	61 (30.4)	183 (24.8)

### Incidence of mortality among asphyxiated neonates

**Fig. 3:**
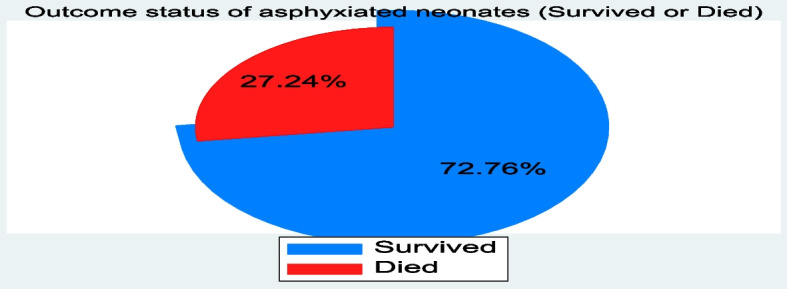
Outcome status of asphyxiated neonates (Survived or Died)

Among the total of 738 neonates with PNA, the cumulative incidence of neonatal mortality among asphyxiated neonates was 201(27.2% (95% CI: 24.1, 30.6)) in the NICU. This is expressed as 272(95% CI: (241,306)) per 1000 admitted neonates with PNA (Fig. 3).

### Significantly associated predictors to mortality among asphyxiated neonates

#### Model development

Twenty-three candidate variables, Socio-demographic, Antepartum, Intrapartum, Neonatal, and clinical predictors, were considered to be predictors of neonatal mortality among asphyxiated neonates admitted to NICU.

On Univariable analysis, maternal age, residence, parity, bad obstetric history, amniotic fluid status, type of pregnancy, gestational age, birth weight, sex of neonate, hypoxic-ischemic encephalopathy, heart rate, temperature, treatment-given, didn’t cry immediately at birth, failure to suck and difficulty of breathing were significantly associated with mortality of asphyxiated neonates at 0.25 cut of point.

Temperature and didn’t cry immediately at birth were significantly associated in the multivariable logistic regression analysis but not significantly in stepwise backward elimination for model reduction. However, in the multivariable logistic regression analysis, and the final reduced model, Residence, bad obstetric history, amniotic fluid status, multiple pregnancy, birth weight (< 2500), hypoxic-ischemic encephalopathy (stage II and III), and failure to suck continued to be significant. Based on these results, a prediction model was developed and an equation for the prediction model was obtained (Table 3).

**Table 3:** Univariable and multivariable analysis for each predictor included in the model and simplified risk score to predict mortality among asphyxiated neonates admitted to NICU at FHCSH, Bahir Dar, Ethiopia, 2021

Predictor Variables	Univariable β (95% CI)	Full Model by Multivariable Analysis		Model Reduction by LR Test		SRS
		β (95% CI)	P–V	P–V	β (95% CI)	
Maternal age						
20–35	0	0	0		0	0
< 20	0.08 (-0.38,0.54)	NA	-		-	-
> 35	0.75 (.05,1.44)	0.52 (-0.39,1.44)	0.263	0.406	NA	-
Residence						
Urban	0	0	0		0	0
Rural	**0.67 (0.34,0.99)**	**0.43 (.03,0.83)**	**0.036**	**0.10**	**0.50 (0.12,0.88)**	**1**
ANC Visit						
Yes	0	0	0		0	0
No	0.09 (-0.70,0.89)	NA	-	-	-	-
Parity						
Multipara	0	0				0
Primipara	0.04 (-0.32,0.40)	NA	-		-	-
G-Multipara	0.74 (0.20,1.27)	0.16 (-0.52,0.84)	0.651	0.626	NA	-
BOH						
No	0	0	0		0	0
Yes	**0.52 (0.13,0.92)**	**0.54 (0.04,1.05)**	**0.035**	**0.160**	**0.60 (0.14,1.06)**	**1**
APH						
No	0	0	0		0	0
Yes	0.09 (-0.50,0.67)	NA	-	-	-	-
PROM						
No	0	0	0		0	0
Yes	0 .11 (-0.31,0.54)	NA	-	-	-	-
Labor Duration						
≤ 18 h	0	0	0		0	0
> 18 h	0.06 (-0.34,0.45)	NA	-	-	-	-
Amniotic Fluid						
Clear	0	0	0		0	0
Stained	**0.59 (0.26,0.92)**	**0.60 (0.20,1.01)**	**0.003**	**0.035**	**0.62 (0.23,1.01)**	**1**
Pregnancy Type						
Single	0	0	0		0	0
Multiple	**1.11 (0.33,1.88)**	**1.26 (0.33,2.20)**	**0.008**	**0.109**	**1.31 (0.39,2.23)**	**3**
Place of Delivery						
Inborn	0	0	0		0	0
Referred	0.03(-0.29,0.36)	NA	-	-	-	-
Gestational Age						
Term	0	0	0		0	0
Preterm	0.65 (0.20,1.10)	0.32 (-0.29,0.92)	0.302		NA	-
Posterm	0.41 (-0.29,1.10)	0.22 (-0.62,1.07)	0.606	0.69	NA	-
Birth Weight						
≥ 2500(g)	0	0	0		0	0
< 2500(g)	**0.66 (0.30,1.03)**	**0.81 (0.31,1.31)**	**0.001**	**0.008**	**0.95 (0.51,1.40)**	**2**
Sex of newborn						
Female	0	0	0		0	0
Male	0 .37 (0.02, 0.73)	0.26 (-0.15,0.67)	0.21	0.59	NA	-
Age at admission						
≤ 6 h	0	0	0		0	0
> 6 h	0.10 (-0.24,0.44)	NA	-	-	-	-
Stage of HIE						
Stage I	0	0				0
Stage II	**0.77 (0.33,1.21)**	**0.87 (0.39,1.34)**	**< 0.001**	**< 0.001**	**0.80 (0.33,1.26)**	**2**
Stage III	**2.54 (2.03,3.05)**	**2.66 (2.09,3.22)**	**< 0.001**		**2.67 (2.12,3.23)**	**5**
Heart Rate (bpm)						
100–160	0	0				0
< 100	0.69 (-0.11,1.48)	0.48 (-0.50,1.47)	0.34	0.70	NA	-
≥ 160	0.09 (-0.54,0.36)	NA	-		-	-
Temperature(^o^c)						
35.5–37.5						
< 35.5	0.60 (0.27,0.94)	0.34 (-0.07,0.74)	0.105	0.55	NA	-
> 37.5	0.13 (-0.94, 0.68)	NA	-		-	-
Sepsis						
No	0	0	0		0	0
Yes	0.02 (-0.32,0.36)	NA	-	-	-	-
Treatment Given						
Yes	0	0	0		0	0
No	-1.33 (-3.40,0.73)	-1.10 (-3.43,1.25)	0.362	0.53	NA	-
Not cry at birth						
No	0	0				0
Yes	0.56 (.18,0.95)	0.37 (-0.11,0.85)	0.134	0.407	NA	-
Failure to suck						
No	0	0	0		0	0
Yes	**0.60 (0.22,0.96)**	**0.58 (0.10,1.06)**	**0.018**	**0.10**	**0.69 (0.26,1.11)**	**1**
Difficulty to Breath						
No	0	0	0		0	0
Yes	0.39 (0.03,0.76)	0.02 (-0.45,0.48)	0.95	0.48	NA	-

Predictors that remained in the reduced final model using the likelihood ratio test are; Residence, bad obstetric history, amniotic fluid status, multiple pregnancy, birth weight (< 2500), HIE (stage II and III), and failure to suck. Stepwise backward elimination for predictor selection was used. NA—not included in the Simplified risk score: we divided the coefficient of predictors included in the reduced model by the smallest (0.43)

*HIE* hypoxic ischemic encephalopathy, *β* beta coefficient, *P–V p*-value, *SRS* simplified risk score, *LR* = Likelihood ratio

⊕ Logit (P) = Log ($\frac{\mathrm{risk of mortality }}{1-\mathrm{risk of mortality}}$) = linear predictor = − 3.11 + 0.50*(Rural Residence) + 0.60*(Bad Obstetric History) + 0.62*(Stained Amniotic Fluid) + 1.31*(Multiple Pregnancy) + 0.95*(Birth weight < 2500 g) + 0.80 (Stage II HIE) + 2.67(Stage III HIE) + 0.69(*(Failure to suck) orby Simplified Risk Score

⊕ Logit (P) = Log ($\frac{\mathrm{risk of mortality }}{1-\mathrm{risk of mortality}}$) = linear predictor = 1*(Rural Residence) + 1*(Bad Obstetric History) + 1*(Stained Amniotic Fluid) + 3*(Multiple Pregnancy) + 2*(Birth weight < 2500 g) + 2*(Stage II HIE) + 5(Stage III HIE) + 1*(Failure to suck)

⇒ Sum of risk score = 1*(Rural Residence) + 1*(Bad Obstetric History) + 1*(Stained Amniotic Fluid) + 3*(Multiple Pregnancy) + 2*(Birth weight < 2500 g) + 2*(Stage II HIE) + 5(Stage III HIE) + 1*(Failure to suck) = 16

Then, we express the probability of mortality by the Bernoulli distribution.

1. Probability of Estimated risk prediction by original β coefficient = 1/(1 + e^−Linear predictor^) = 1/ (1 + e – (− 3.11 + 0.50*(Rural Residence) + 0.60*(Bad Obstetric History) + 0.62*(Stained Amniotic Fluid) + 1.31*(Multiple Pregnancy) + 0.95*(Birth weight < 2500 g) + 0.80 (Stage II HIE) + 2.67(Stage III HIE) + 0.69(*(Failure to suck)).
2. Probability of Estimated risk prediction by simplified risk score = 1/(1 + e^−Linear predictor^) = 1/ (1 + e—(1*(Rural Residence) + 1*(Bad Obstetric History) + 1*(Stained Amniotic Fluid) + 3*(Multiple Pregnancy) + 2*(Birth weight < 2500 g) + 2(Stage II HIE) + 5(Stage III HIE) + 1*(Failure to suck)) [37–41].

#### Model performance, calibration and validation

**Fig. 4:**
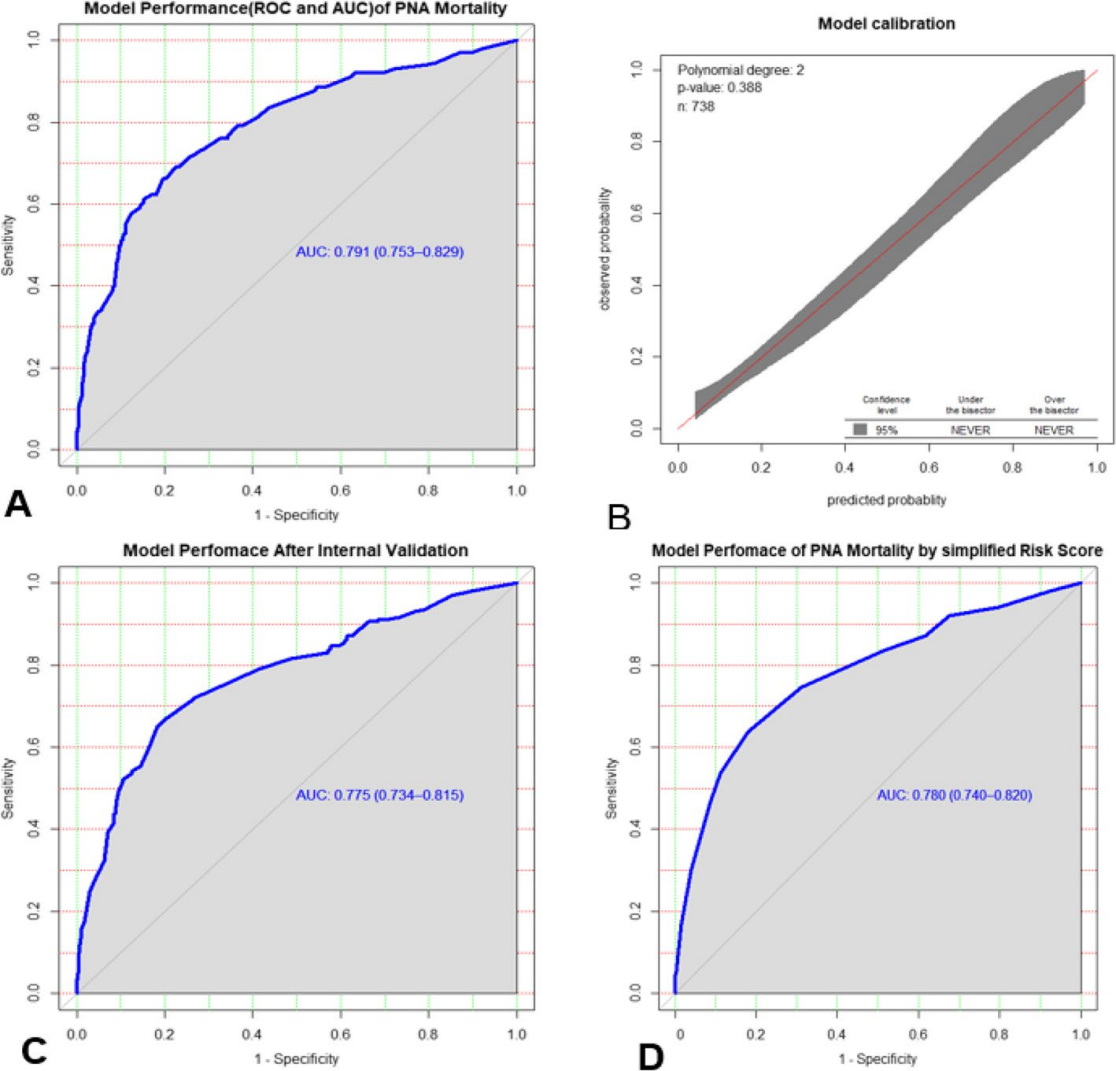
**A** Area under the ROC curve for original prediction model, and **B** predicted versus observed probability of Mortality among asphyxiated neonates, **C** Area under the ROC curve after internal validation and **D** Area under the ROC curve by simplified risk score

The Area under the ROC curve of the final reduced model was 0.791 (95% CI: 0.753–0.829). The goodness of fit test for the model was checked by both Hosmer-Lemshow and the calibration plot. The calibration test had a *p*-value of 0.388 and the Hosmer-Lemshow goodness of fit test showed a *p*-value = 0.81, indicating that the model does not misrepresent the data. The validation of the model using the bootstrap sampling technique showed less optimism with an optimism coefficient of 0.016, leading to a corrected performance of the model (AUC) of 0.775 (corrected 95% CI: 0.734–0.815) (Fig. 4).

#### Density plot to show the ability of the model to separate survived and died

**Fig. 5:**
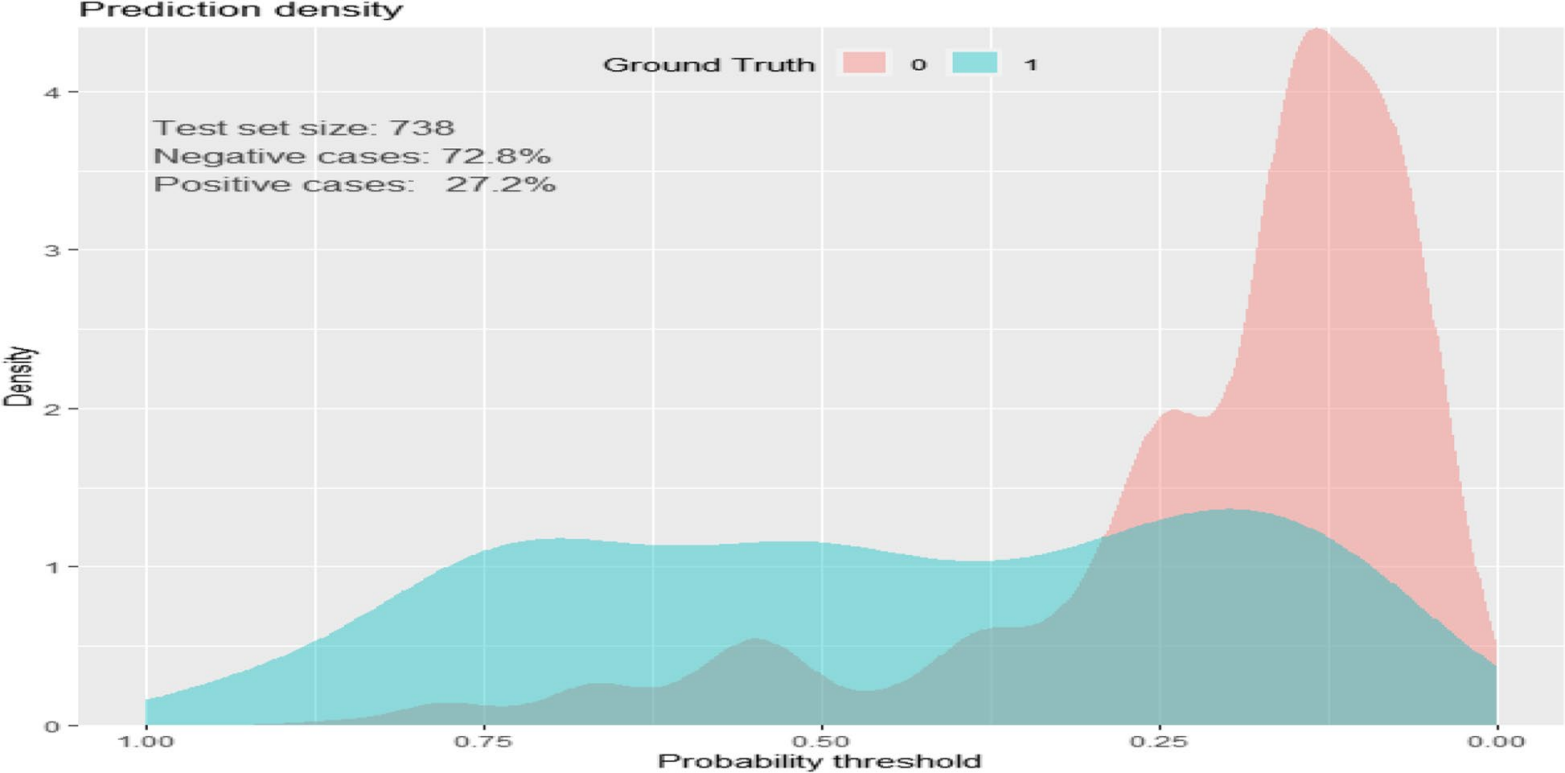
Density plot showing model performance of a developed risk score (how it sensitive and specific)

As shown in the Fig. 5 below, the density plot shows the model performance of a developed risk score. That means how the model separates died and survived neonates with PNA. 27.2% of asphyxiated neonates level as “1” who died and 72.8% of asphyxiated neonates level as “0” who survived during 28 days of follow-up at NICU. Even though there is an overlap between died and survived neonates, the model shows good insight to identify died and survived asphyxiated neonates (Fig. 5).

#### Simplified risk score development

**Fig. 6:**
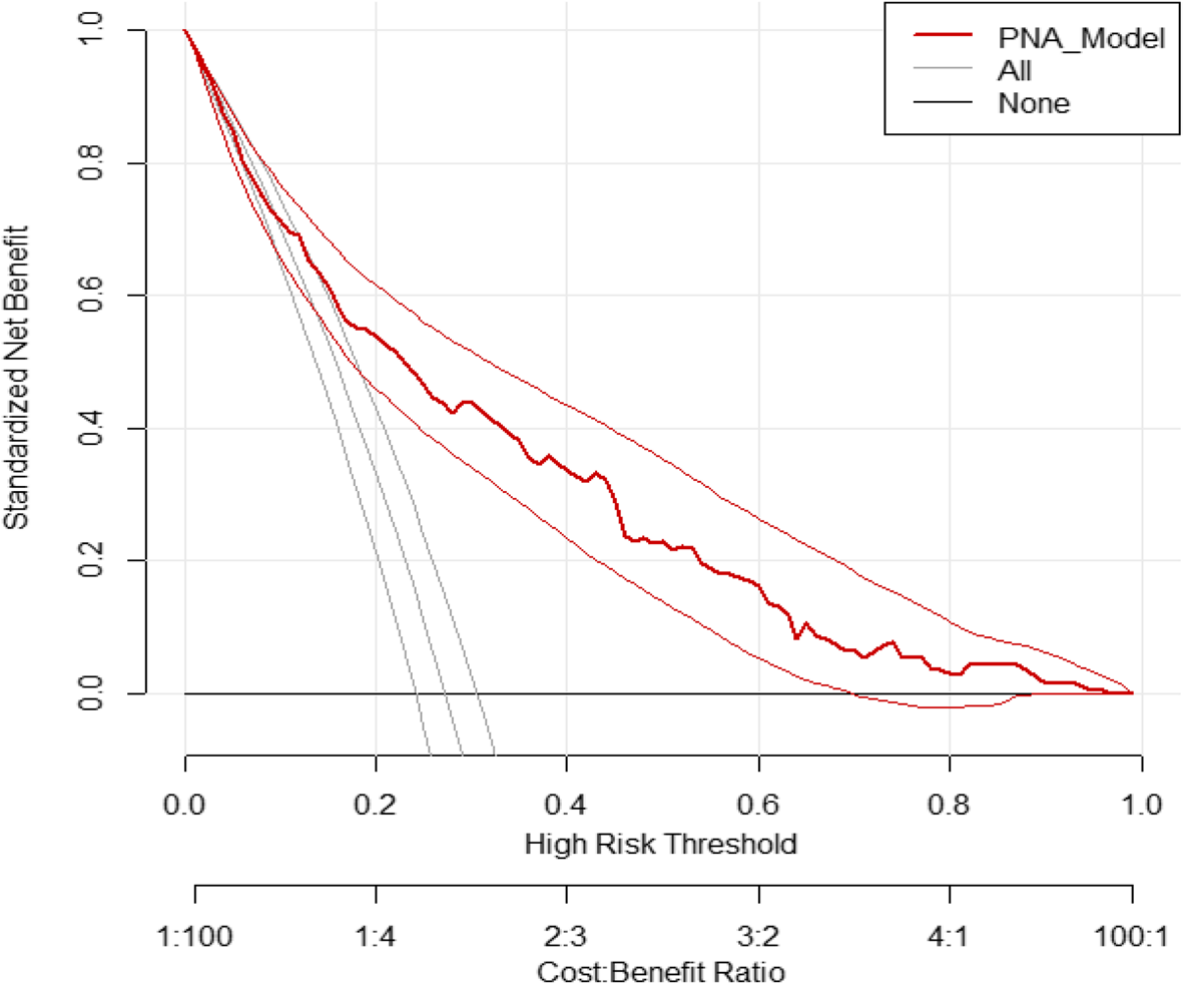
A decision curve. Plotting net benefit of the model verses threshold probability and corresponding cost–benefit ratio

To make it easy for the practical application, we developed a simplified risk score from the final reduced model or significant β coefficients. Dividing of significant β coefficients by the lowest significant β coefficient and Rounding to the nearest integer resulted in a simplified prediction score. The chance of minimum and maximum risk scores of mortalities among asphyxiated neonate can range from 0 to 16 (Table 3). The simplified risk score had comparable prediction accuracy with the original β coefficients, with an AUC of 0.78 (95% CI 0.74 to 0.82) (Fig. 6).

#### Risk classification of mortality among asphyxiated neonates using simplified risk score

Based on the “Youden Index” an optimal cutoff point for Simplified Risk Score was ≥ 7. With this cutoff, the sensitivity, specificity, positive predictive value, negative predictive value, positive likelihood ratio, and negative likelihood ratio were 0.64, 0.82, 0.57, 0.86, 3.56, and 0.44 orderly.

PPV is the ratio of patients truly Predicted having of mortality to all those who had positively Predicted results (including patients with or without future mortality). This can predict how likely it is for someone to truly be have death, in case of a positive predicted result. In our study the PPV of a prediction for the future mortality is 57%, it means 57% of patient who predicted positive actually will have mortality in the future (Table 4).

**Table 4:** Sensitivity, specificity PPV, NPV, LR^+^, and LR^−^ of the model for Mortality among asphyxiated neonates by Simplified Risk scores at different cutoff points

Cutoff	High Risk n (%)	Sensitivity	Specificity	PPV	NPV	LR^+^	LR^−^
≥ 5	493(66.8)	0.84	0.48	0.38	0.89	1.62	0.33
≥ 6	439(59.5)	0.75	0.69	0.47	0.88	2.42	0.36
≥ 7	331(44.8)	0.64	0.82	0.57	0.86	3.56	0.44
≥ 8	256(37.7)	0.53	0.89	0.64	0.84	4.82	0.53
≥ 9	188(25.5)	0.47	0.91	0.67	0.82	5.22	0.58

*PPV* Positive predictive value, *NPV* negative predictive value, *LR*^+^ Positive likelihood ratio, *LR*^*−*^ = negative likelihood ratio [37]

Based on this model asphyxiated neonates can have a risk score of mortality of 0 and 16 respectively. The Incidence of Mortality among Asphyxiated Neonates were 11.3%, 21.7%, and 64.3%, respectively, in the low (score < 5), medium (5 to 7), and high-risk group (> 7) [37] (Table 5).

**Table 5:** Risk classification of mortality among asphyxiated neonates using simplified prediction score

Risk stratification	Asphyxiated Neonates n (%)	Incidence of Mortality n (%)
Low Risk (< 5)	293(39.5%)	33(11.3%)
Moderate Risk (5–7)	277(37.5%)	60(21.7%)
High Risk (> 7)	168(22.8)	108(64.3%)
Total	738(100%)	201(27.2%)

#### Decision curve analysis

Across the whole range of threshold probabilities, the model has the highest net benefit, indicating that it has the best clinical and public health value. Therefore, utilizing the model to improve health care service decisions offers a good net benefit than treating in the usual way regardless of their risk threshold (Fig. 6).

## Discussion

In this study, the cumulative incidence and incidence rate of mortality among asphyxiated neonates was 27.2% (95% CI: 24.1, 30.6) and 35.8 (95% CI: (31.2, 41.2)) per 1000 neonatal admission –days respectively. In the present study, we developed a simplified risk prediction score to screen the risk of mortality among asphyxiated neonates during admission to NICU with the discrimination power (AUC) of 0.78 (95% CI 0.74 to 0.82).

Based on the “Youden Index” an optimal cutoff point for Simplified Risk Score was ≥ 7. With this cutoff, the sensitivity, specificity, positive predictive value, negative predictive value, positive likelihood ratio, and negative likelihood ratio were 0.64, 0.82, 0.57, 0.86, 3.56, and 0.44 orderly. Sensitivity = 64% and Specificity = 82% indicate the concordance of our prediction score with respect to true mortality among asphyxiated neonates. While PPV = 57% and NPV = 86% respectively indicate the likelihood that our prediction score can successfully identify whether people do or do not have mortality.

The Combination of predictors to develop a simplified prediction score in the final reduced model were Residence, bad obstetric history, amniotic fluid status, multiple pregnancy, birth weight (< 2500 g), hypoxic-ischemic encephalopathy (stage II and III), and failure to suck.

The finding of this study (27.2%) is almost comparable with the study conducted in Northern Nigeria-28% [31]. The agreement of these results might be due to the same higher-level hospitals and institutional-based secondary data were studied in both hospitals in the neonatal intensive care unit. The current finding is higher than studies done in South Africa-13.3% [42], South-East Nigeria-18% [30], Tanzania-23% [29], and Southern Kerala-25.8% [43]. The difference between our study and the study done in South Africa and other settings may be country-based maternal and neonatal care systems, hospital setup, socio-demographic, nutritional, and other cultural situations. However, the result of the present study demonstrated that the incidence of Neonatal Mortality among asphyxiated neonates is lower than the result of the study done in India among referred neonates to the NICU-40.6% [44]. All out born referral neonates diagnosed with perinatal asphyxia were included in the Indian study, but our study included both inborn and referral neonates with perinatal asphyxia.

Predicting the probability of mortality among asphyxiated neonates is important to take appropriate intervention accordingly. In our study, the discriminatory power of the simplified risk prediction score in the final reduced model was quantified by an AUC of 0.78 (95% CI 0.74 to 0.82), which is the acceptable discriminatory power, and the model performed well [45]. The performance ability (AUC = 0.78) of the simplified risk prediction score in this model is consistent with the performance ability (AUC = 0.791) from the original beta coefficient. It is also in line with the performance power (0.775) of the model after internal validation by using the bootstrap resampling technique showed less optimism with an optimism coefficient of 0.016.

Based on the “Youden” index having ≥ 7 as a cutoff point, our prediction score has an optimal level of specificity, sensitivity, PPV, NPV LR^+^ and LR^−^ 0.64, 0.82, 0.57, 0.86, 3.56, and 0.44 orderly to predict mortality among asphyxiated neonates. It is a trade of measurement. We can change the cutoff point to increase either of the accuracy measures at the expense of another accuracy. Since mortality and morbidity among asphyxiated neonates is a severe clinical and public health problems it is better to decrease the cutoff to increase sensitivity rather than specificity depending on the program aim and availability of resources.

Previous research aimed to explain Risk factors for mortality in asphyxiated neonates and estimate the effect sizes like odds ratio in developed and developing countries. In recent years, the emphasis has changed on predicting mortality among asphyxiated neonates using a combination of prognostic factors with discrimination ability.

Our final reduced risk score model was built 7 prognostic factors to predict mortality among asphyxiated neonates: Residence, bad obstetric history, amniotic fluid status, multiple pregnancy, birth weight (< 2500 g), hypoxic-ischemic encephalopathy (stage II and III), and failure to suck.

Similar to existing evidence [18] and the findings from South-East Nigeria [30], India [44], and Southern Kerala [43] studies, the current study demonstrated that the severity stage of HIE was the strongest predictor of mortality among asphyxiated neonates. This is due to physiologic and biochemical derangement of hypoxic and ischemic neonates resulting in acquired brain injury and mortality.

Stained amniotic fluid was a significant discriminatory prognostic factor of neonatal mortality with PNA. Supported by the study conducted in Northern-Central Nigeria [31]. This is explained by meconium-stained amniotic fluid or liquor is associated with respiratory distress and meconium aspiration syndrome before delivery and immediately after delivery resulting in perinatal asphyxia and its mortality. The presence of meconium-stained amniotic fluid is an early warring condition that needs closer follow-up and monitoring of mothers and their newborns.

In this study, BOH and multiple pregnancy had associated with mortality among asphyxiated neonates. In line with our study, the Population-Based Nested Case–Control Study in West Gojam showed that previous history of perinatal death, abortion, and twin birth were significantly associated with perinatal mortality [46]. A possible explanation might be improper newborn handling practices which prevail in the community for ages. The need to have another baby sooner to replace the lost baby immediately leads to narrow birth spacing which increases the risk of newborn death. Abortion is one of the major causes of bleeding in the first trimester and resulted in preterm birth, low birth weight, and perinatal mortality [47]. Multiple pregnancy may end up in prolonged labor, birth trauma, antepartum hemorrhage, and other intrapartum complications [47].

According to the national strategy for newborn and child survival in Ethiopia from 2015/16–2019/20 rural residence is strongly associated with neonatal mortality [12]. Likewise, our study also indicated that rural residence was associated to discriminate the probability of mortality among asphyxiated neonates. This might be explained by several reasons like health service accessibility, distance from the health facility, awareness level, ambulance availability, timely referral linkage, the capacity of health providers in rural health facilities, sanitation, and nutrition all can be related to mothers living in the rural area.

This study showed that low birth weight (< 2500 g) is one of the predictors associated with discriminating the likelihood of mortality among asphyxiated neonates. This is also supported by the study conducted in a Tanzanian rural hospital to know a major cause of early neonatal mortality. The possible explanation might be low birth weight, increases neonatal morbidity, and mortality. It is linked to neonatal and childhood health outcomes like infection susceptibility, neurological impairments, and poor cognitive abilities. In long run, it is also linked to high blood pressure, diabetes, and coronary heart disease later in life [14, 18].

Our study demonstrated that failure to suck breastfeeding had been associated with mortality among asphyxiated neonates. This is also supported by the study conducted in low-resource settings to determine factors associated with mortality among asphyxiated newborns [29] and Nelson Textbook of Pediatrics 21 EDITION [18]. The possible explanation is the development of sucking and swallowing in the newborn, in coordination with breathing, are essential for safe and successful oral feeding. Failure to suck can occur from a disruption of these coordinated activities, increasing the risk of apnea, bradycardia, failure to thrive, oxygen desaturation, and finally mortality. As a result, identifying infants who are at risk for sucking and swallowing problems is critical to avoid feeding problems and potentially serious complications, and finally mortality.

To the best of our knowledge, this is the first study that develops an early warning risk prediction score for neonatal mortality with perinatal asphyxia in LMIC including Ethiopia, and our study setting FHCSH from easily obtainable and applicable prognostic factors. To provide information on the combined performance of prognostic factors, a risk prediction score was built to predict mortality among asphyxiated neonates by using Transparent Reporting of a multivariable prediction model for Individual Prognosis or Diagnosis (TRIPOD) statement [48].

We develop this simplified risk scores in the final reduced model using easily accessible Predictors by history and physical examination for any health professionals without sophisticated laboratory and imaging investigation. The simplified risk prediction score developed using simple integer this is easier to use in ordinary clinical and public health practice.

The other added value of this prognostic risk prediction score in the decision curve analysis revealed that across the whole range of risk/threshold probabilities the model has a high net benefit. Indicating that it has the best clinical and public health value.

The strengths of the study. Firstly, our prediction model is constructed from easily obtainable characteristics that make it applicable. Secondly, we internally validated our model using the 10000 bootstrap resampling technique and resulted in a minimal optimism coefficient, indicating our model is less sample-dependent. Thirdly, we built a multivariable risk prediction prognostic score for the individual patient using a sufficient number of participants and predictors based on the TRIPOD Statement rather than univariable.

However, this study might have the following limitations. Firstly, since the data was collected retrospectively, all relevant predictors that may not be available in the original records, which might have a good discrimination power. Enthought AUC (0.78) of risk prediction has acceptable discriminatory power AUC (0.78) it is better to increase. Secondly, even though the bootstrapping showed minimal optimism and indicating a stable predictive capability of the model, due to small sample size, we did not validate the model in separate training and test datasets. Lastly, because of single site study, which needs external validation such as geographic validation in different facilities, Time validation and context validation before application it in Health facilities.

## Conclusion

The incidence of mortality among asphyxiated neonates (27.2%,) in this study setting was high from the national figure (24%), and SDG targets to end preventable newborn death and stillbirths by 2030. We developed a simplified risk score with good discrimination power using a Combination of rural residence, bad obstetric history, stained amniotic fluid status, multiple pregnancy, birth weight (< 2500 g), hypoxic-ischemic encephalopathy (stage II and III), and failure to suck predictors. The risk score developed from this study helps to identify neonates with perinatal asphyxia that are at higher risk of mortality. It also alerts for treatment prioritization upon NICU admission and guides decisions about resource allocations. Hence, this feasible prediction score would offer an opportunity to reduce neonatal mortality related to PNA and improve the overall neonatal health care utility.

### Recommendation

#### Ministry of health and health bureau

In this study hypoxic-ischemic encephalopathy is the strongest predictor of neonatal mortality with PNA, which needs therapeutic hypothermia and other advanced treatment. And as we have seen during the data collection period, the number of senior physicians in FHCSH is below the standard. Therefore, the Ministry of Health and Health Bureau should give attention to increase the number of trained senior physicians and to fulfill NICU service equipment.

#### Hospital

To increase and improve critical care units, and train manpower according to standard neonatal and maternal care guidelines. To encourage and follow health professionals to apply this simple to use risk prediction score.

#### Community

To improve timely health-seeking behavior to prevent problems of BOH and multiple pregnancy-related problems and to prevent rural risk factors (sanitation, nutrition).

#### Health professionals

Timely diagnosis and referring mothers with multiple pregnancies and bad obstetric history. For health providers who work in primary health care units.

Timely diagnosis and referring of high-risk neonates. More emphasis on neonates with HIE, failure to suck, birth weight less than 2500 g, and stained amniotic fluid. Utilize this simple risk score and careful case management based on the risk.

#### Researchers

Before introducing this risk prediction score to clinical and public health practices in other settings, we recommend external validation for transferability.

We also recommend further study should be studied with prospective real-world data which is collected for research purposes.

### Supplementary Information

**Supplementary Material 1.** 

**Supplementary Material 2.** 

## Data Availability

All data generated or analyzed during this current study are included in this article and its supplementary information files.

## Abbreviations

ANC: Ante-Natal Care
APH: Antepartum Hemorrhage
APGAR: Appearance Pulse Grimace Activity Respiration
AUC: Area Under Curve BOH: Bad Obstetric History
CI: Confidence Interval
FHCSH: Felege-Hiwot Comprehensive Specialized Hospital
EDHS: Ethiopia Demographic Health Survey
HIE: Hypoxic-Ischemic Encephalopathy HR: Heart Rate
LMIC: Low and Middle-Income Countries
NICU: Neonatal Intensive Care Unit
PNA: Peri-Natal Asphyxia
PROM: Premature Rupture of Membrane
ROC: Receiver Operator Characteristics
WHO: World Health Organization

## References

[1] Organization, W.H. (2012). Guidelines on basic newborn resuscitation.

[2] Manu A (2018). Assessment of facility readiness for implementing the WHO/UNICEF standards for improving quality of maternal and newborn care in health facilities–experiences from UNICEF’s implementation in three countries of South Asia and sub-Saharan Africa. BMC Health Serv Res.

[3] Lindbäck C (2014). Poor adherence to neonatal resuscitation guidelines exposed; an observational study using camera surveillance at a tertiary hospital in Nepal. BMC Pediatr.

[4] Carter BS, Haverkamp AD, Merenstein GB (1993). the definition of acute perinatal asphyxia. Clin Perinatol.

[5] Ahearne CE, Boylan GB, Murray DM (2016). Short and long term prognosis in perinatal asphyxia: An update. World J Clin Pediatr.

[6] McClure EM (2018). Global network for women's and children's health research: probable causes of stillbirth in low-and middle-income countries using a prospectively defined classification system. BJOG.

[7] Odd D (2017). Hypoxic-ischemic brain injury: Planned delivery before intrapartum events. J Neonatal Perinatal Med.

[8] Lawn JE, Wilczynska-Ketende K, Cousens SN (2006). Estimating the causes of 4 million neonatal deaths in the year 2000. Int J Epidemiol.

[9] Lawn J (2009). Newborn survival in low resource settings—are we delivering?. BJOG.

[10] Robertson NJ (2011). Pilot randomized trial of therapeutic hypothermia with serial cranial ultrasound and 18–22 month follow-up for neonatal encephalopathy in a low resource hospital setting in Uganda: study protocol. Trials.

[11] Ahmed I (2018). Population-based rates, timing, and causes of maternal deaths, stillbirths, and neonatal deaths in south Asia and sub-Saharan Africa: a multi-country prospective cohort study. Lancet Glob Health.

[12] Ethiopia Federal Ministry of Health National strategy for newborn and child survival in Ethiopia: 2015/16–2019/20. Ethiopia Federal Ministry of Health Addis Ababa, Ethiopia; 2015.

[13] Liu L (2015). Global, regional, and national causes of child mortality in 2000–13, with projections to inform post-2015 priorities: an updated systematic analysis. Lancet.

[14] Central Statistical Agency/CSA/Ethiopia and ICF (2016). Ethiopia demographic and health survey 2016.

[15] UNICEF, U.J.N.Y.U. (2015). Levels and trends in child mortality.

[16] Malta DC, Duarte EC, Almeida MF, Dias MA, Morais Neto OL, Moura LD, Ferraz W, Souza MD (2007). List of avoidable causes of deaths due to interventions of the Brazilian Health System. Epidemiol Serv Saúde..

[17] Pitsawong C, Panichkul P. Risk factors associated with birth asphyxia in Phramongkutklao Hospital. Thai J Obstet Gynecol. 2011;19:165–71.

[18] Kliegman RM, Behrman RE, Jenson HB, Stanton BM. Nelson textbook of pediatrics e-book. Elsevier Health Sciences; 2007.

[19] Kubben P, Dumontier M, Dekker A (2019). Fundamentals of clinical data science.

[20] Riley RD (2019). Minimum sample size for developing a multivariable prediction model: PART II-binary and time-to-event outcomes. Stat Med.

[21] Houweling TA (2019). A prediction model for neonatal mortality in low-and middle-income countries: an analysis of data from population surveillance sites in India, Nepal and Bangladesh. Int J Epidemiol.

[22] Márquez-González H (2015). Development and validation of the neonatal mortality score-9 Mexico to predict mortality in critically ill neonates. Arch Argent Pediatr.

[23] Colglazier W (2015). Sustainable development agenda: 2030. Science.

[24] Miller NP (2014). Integrated community case management of childhood illness in Ethiopia: implementation strength and quality of care. Am J Trop Med Hyg.

[25] Ethiopian Public Health Institute (EPHI) [Ethiopia] and ICF. Ethiopia Mini Demographic and Health Survey 2019: Final Report. Rockville: EPHI and ICF; 2021.

[26] Hassan ZA, Schattner P, Mazza D (2006). Doing a pilot study: why is it essential?. Malays Fam Physician.

[27] Shetty DK, Alzhrani DA, Alotaibi MK, Alotaibi SM, Almaghrabi RY (2020). Comparative evaluation of gingival displacement by using retraction paste and retraction cord-in-vivo pilot study. Int J Life Sci Pharma Res.

[28] Kent P, Cancelliere C, Boyle E, Cassidy JD, Kongsted A (2020). A conceptual framework for prognostic research. BMC Med Res Methodol..

[29] Cavallin F, Menga A, Brasili L, Maziku D, Azzimonti G, Putoto G, Trevisanuto D (2022). Factors associated with mortality among asphyxiated newborns in a low-resource setting. J Matern Fetal Neonatal Med..

[30] Ekwochi U (2017). Incidence and predictors of mortality among newborns with perinatal asphyxia: a 4-year prospective study of newborns delivered in health care facilities in Enugu South-East Nigeria. Clin Med Insights Pediatr.

[31] Ige OO (2013). Risk factors and mortality rate of severely asphyxiated neonates in a tertiary centre in north-central Nigeria. Jos J Med.

[32] Kawakami MD (2021). Neonatal mortality associated with perinatal asphyxia: a population-based study in a middle-income country. BMC Pregnancy Childbirth.

[33] Wang Q (2007). Determination of the selection statistics and best significance level in backward stepwise logistic regression. Commun Stat Simul Comput.

[34] Steyerberg EW (2000). Prognostic modelling with logistic regression analysis: a comparison of selection and estimation methods in small data sets. Stat Med.

[35] Efron B, Tibshirani RJ. An introduction to the bootstrap: CRC press. Ekman, P., & Friesen, WV (1978). Manual for the facial action coding system. 1994.

[36] Zheng M (2022). Tumor mutation burden for predicting immune checkpoint blockade response: the more, the better. J Immunother Cancer.

[37] Hassen HY (2020). Development and validation of a risk score to predict low birthweight using characteristics of the mother: analysis from BUNMAP cohort in Ethiopia. J Clin Med.

[38] Janssen KJ (2009). A simple method to adjust clinical prediction models to local circumstances. Can J Anaesth.

[39] Muche AA, Olayemi OO, Gete YK (2020). Predictors of postpartum glucose intolerance in women with gestational diabetes mellitus: a prospective cohort study in Ethiopia based on the updated diagnostic criteria. BMJ Open.

[40] Bos JM (2018). Prediction of clinically relevant adverse drug events in surgical patients. PLoS One.

[41] Grobbee DE, Hoes AW. Clinical epidemiology: principles, methods, and applications for clinical research. Jones & Bartlett Publishers; 2014.

[42] Padayachee N, Ballot DE (2013). Outcomes of neonates with perinatal asphyxia at a tertiary academic hospital in Johannesburg. South Africa. The SAJCH.

[43] Joseph S (2017). Clinical profile and short-term outcome of perinatally asphyxiated term neonates in a tertiary hospital in Southern Kerala. IJCH.

[44] Meshram RM, Bokade C (2019). Risk factors for mortality in birth asphyxia of outborn neonates: a prospective observational study. Sri Lanka J Child Health.

[45] Meshram RM, Bokade C (2019). Risk factors for mortality in birth asphyxia of outborn neonates: a prospective observational study. Sri Lanka J Child Health.

[46] Yirgu R (2016). Perinatal mortality magnitude, determinants and causes in west gojam: population-based nested case-control study. PLoS One.

[47] Federal Ministry of Health, Management protocol on selected obstetrics topics. Federal Democratic Republic of Ethiopia; 2010.

[48] Collins GS (2015). Transparent reporting of a multivariable prediction model for individual prognosis or diagnosis (TRIPOD): the TRIPOD statement. Br J Surg.

