# Supplementary material for: Incidence and development of validated mortality prediction model among asphyxiated neonates admitted to neonatal intensive care unit at Felege Hiwot Comprehensive Specialized Hospital, Bahir Dar, Northwest Ethiopia, 2021: retrospective follow-up study
Source: BMC Pediatr. 2024 Mar 28;24:219. doi: 10.1186/s12887-024-04696-0 (PMC10976726; doi:10.1186/s12887-024-04696-0)
Supplement: Supplementary file 1 — Supplementary Material 1. [file 12887_2024_4696_MOESM1_ESM.docx]

Supplementary Table 1: Distribution of Missing value and observed cases among Predictors of Mortality in Neonates with Perinatal Asphyxia Admitted to NICU at FHCSH, Bahir Dar, Ethiopia, 2021

|  | Missing | | Valid  N |
| --- | --- | --- | --- |
|  | N | Percent |  |
| Duration of labor | 78 | 10.6% | 660 |
| Premature rupture of membrane (PROM) | 38 | 5.1% | 700 |
| Antepartum hemorrhage (APH) | 37 | 5.0% | 701 |
| Gestational age | 35 | 4.7% | 703 |
| Birth weight | 34 | 4.6% | 704 |
| Age of neonate | 33 | 4.5% | 705 |
| Amniotic Fluid Status | 31 | 4.2% | 707 |
| Type of Pregnancy | 20 | 2.7% | 718 |
| Bad Obstetric History | 19 | 2.6% | 719 |
| Severity Stage of HIE | 17 | 2.3% | 721 |
| Sepsis | 14 | 1.9% | 724 |
| Place of delivery | 12 | 1.6% | 726 |
| Parity | 12 | 1.6% | 726 |
| Had ANC visit | 10 | 1.4% | 728 |
| Temperature | 7 | 0.9% | 731 |
| Heartrate | 7 | 0.9% | 731 |
| Treatment given | 6 | 0.8% | 732 |
| Maternal Age | 2 | 0.3% | 736 |
| Residence of mother | 2 | 0.3% | 736 |
| Sex of newborn | 1 | 0.1% | 737 |
| Difficulty of Breathing | 0 | 0.0% | 738 |
| Failure to suck | 0 | 0.0% | 738 |
| Did not cry at birth | 0 | 0.0% | 738 |
